# Alpha‐actinin‐4 is essential for maintaining normal trophoblast proliferation and differentiation during early pregnancy

**DOI:** 10.1186/s12958-021-00733-0

**Published:** 2021-03-23

**Authors:** Wei Peng, Ying Liu, Hongbo Qi, Qingshu Li

**Affiliations:** 1 Department of Obstetrics, The First Affiliated Hospital of Chongqing Medical University, 400016 Chongqing, China; 2 Chongqing Key Laboratory of Maternal and Fetal Medicine, Chongqing Medical University, 400016 Chongqing, China; 3 Joint International Research Laboratory of Reproduction and Development of Chinese Ministry of Education, Chongqing Medical University, 400016 Chongqing, China; 4 Department of Pathology, School of Basic Medicine, Chongqing Medical University, 1 Yixueyuan Rd, Yuzhong District, 400016 Chongqing, China

**Keywords:** ACTN4, Trophoblast, Proliferation, Invasion and migration, Preeclampsia

## Abstract

**Background:**

Proper differentiation of trophoblasts in the human placenta is essential for a successful pregnancy, whereas abnormal regulation of this process may lead to adverse pregnancy outcomes, especially preeclampsia (PE). However, the underlying mechanism of trophoblast differentiation remains unclear. Previous studies have reported the involvement of alpha-actinin-4 (ACTN4) in the actin cytoskeleton dynamics and motility. Hence, we hypothesized that ACTN4 may act as an important regulator in the normal proliferation and differentiation of trophoblasts during early pregnancy.

**Method:**

To test this hypothesis, we collected villous tissues from women undergoing a legal pregnancy termination during 6–10 weeks of gestation and explanted them for cell culture and siRNA transfection. We also obtained placental tissues from PE patients and healthy pregnant women and isolated the primary cytotrophoblast (CTB) cells. The expression of ACTN4 in the CTBs of placental villi and during the differentiation of CTBs into STBs was detected by immunofluorescence, immunohistochemistry (IHC), and EdU proliferation assays. Besides, villous explant, Matrigel invasion, transwell migration assay, and Wound-healing assay were performed to identify the possible role of ACTN4 in the outgrowth of explants and the invasion, migration, and proliferation of cell column trophoblasts (CCTs). Western blot analysis was carried out to compare the protein expression level of AKT, Snail activities, and epithelial-to-mesenchymal transition (EMT) in the villi or HTR8/SVneo cells with ACTN4 knockdown.

**Results:**

ACTN4 was highly expressed in CTB cells and interstitial extravillous trophoblast (iEVT) cells but not found in the syncytiotrophoblast (STB) cells in the first trimester villi. Downregulation of ACTN4 led to reduced trophoblast proliferation and explant outgrowth *ex vivo*, as well as iEVT invasion and migration *in vitro* due to disrupt of actin filaments organization. Such ACTN4 inhibition also decreased AKT and Snail activities and further impeded the EMT process. In addition, ACTN4 expression was found to be downregulated in the iEVTs from preeclamptic placentas.

**Conclusions:**

Our findings suggest that ACTN4 may act as an important regulator of trophoblast proliferation and differentiation during early pregnancy, and dysregulation of this protein may contribute to preeclampsia pathogenesis.

## Background

Normal human placental development during the first trimester is considered critical for embryonic survival and maintenance of a healthy pregnancy. The basic structural and functional unit of the placenta is the placental villus [1]. Cytotrophoblast (CTB) progenitors are a layer of mononucleated cells lining the inner portion of the floating placental villi, which can further differentiate into a layer of multinucleated syncytiotrophoblast (STB) cells. CTB cells are known to be implicated in regulating nutrient, water, waste, and gas exchanges between maternal and fetal circulations and producing various hormones vital for fetal development and maintenance of normal pregnancy [2]. Besides, when undergoing an epithelial-to-mesenchymal transition (EMT), proliferative CTBs of the anchoring villi differentiate into extravillous trophoblasts (EVT), thus acquiring a typical motility phenotype to allow for invasion into the maternal uterus [3, 4]. Those trophoblast cells invading the maternal decidua and anchoring the placenta are called interstitial extravillous trophoblasts (iEVTs) [5]. Defective CTB differentiation, particularly in the cases of limited iEVT invasion into the decidua and ensuing failures in remodeling maternal spiral arteries, has been thought to lead to preeclampsia (PE), which is a highly prevalent pregnancy-related complication marked by new-onset hypertension and proteinuria after 20 weeks of gestation resulting in maternal and perinatal morbidity and mortality [6, 7].

EMT is a well-orchestrated process that facilitates invasion through attenuating cell-cell adhesion, reorganizing the actin cytoskeleton, and upregulating the expression of mesenchymal markers [8–10]. Cell motility and invasion are essential activities involved in the dramatic changes in cellular morphology associated with dynamic cytoskeleton remodeling [11]. Importantly, successful cellular invasion depends on the emergence of invasive structures [12, 13], suggesting that cytoskeletal integrity is critical for EMT-induced migration and invasion of EVTs.

Alpha-actinins (ACTNs) are known to maintain the cytoskeletal structure and modulate cell motility [14]. Among the four members of the ACTN family in humans, ACTN2 and ACTN3 are muscle-specific and are predominantly localized in the Z lines of striated muscle cells, while ACTN1 and ACTN4 are ubiquitously expressed. All these actinins share high sequence homology. In contrast to ACTN1, which is mainly expressed at cell-cell adherens junctions, ACTN4 is predominantly found at the leading edge of moving cells [15, 16], suggesting that ACTN4 may be involved in cell migration. In addition, ACTN4 signaling connects integrin with the actin cytoskeleton and potentiates trophoblast invasion in bovine placentas [17]. A recent investigation revealed that ACTN4 is highly expressed in EVTs based on the microarray data from the gene expression omnibus database [18]. Furthermore, ACTN4 deficiency has been reported to dramatically attenuate the proliferation and invasion of various cancer cells [19–21]. The accumulating evidence strongly implies that ACTN4 may participate in trophoblast proliferation and invasion. However, the true functions of ACTN4 in trophoblast and placental development remain obscure.

Given the role of this protein in the actin cytoskeleton dynamics and motility, we hypothesized that ACTN4 is crucial for successful trophoblast invasion and migration, and ACTN4 deficiency may ultimately contribute to poor placentation and PE development.

## Materials and Methods

### Sample collection

Villous tissues were collected from women who underwent a legal pregnancy termination for non-medical reasons at the first trimester of gestation (6–10 weeks). Those with a history of spontaneous abortion or ectopic pregnancy were excluded. Placental tissues were obtained at the maternal side from women with normal pregnancies and patients with PE (defined as a new onset of hypertension (systolic/diastolic blood pressure ≥ 160/110 mmHg measured on two occasions at least 4 h apart) and proteinuria after 20 weeks of gestation) during cesarean deliveries. Patients with other pregnancy-related complications were also excluded. A portion of biopsies was immediately snap frozen in liquid nitrogen and stored at − 80 °C for later use, while the remaining tissues were fixed in 4 % paraformaldehyde and then embedded in paraffin. The patients’ clinical features are summarized in Table 1. For all samples, written informed consent was obtained, and this research was approved by the Ethics Committee of the First Affiliated Hospital of Chongqing Medical University.

**Table 1:** Clinical characteristics of the subjects

Characteristics	Normal pregnancy (*n* = 8)	sPE (*n* = 7)	*p*
Maternal age (years)	25 (22–31)	28 (18–32)	0.715
BMI (kg/m^2^)	29.1 (21.6–35.1)	30.5 (23.6–37.3)	0.694
Systolic blood pressure (mmHg)	111.5 (99–125)	163 (150–166)	0.003
Diastolic blood pressure (mmHg)	69.5 (56–78)	105 (99–110)	0.003
Proteinuria (-/+)	- ~ +	+ ~ ++++	-
Gestational day at delivery (days)	282 (274–289)	270 (258–284)	0.036
Neonatal birth weight (g)	3665 (3300–4360)	3380 (2620–3600)	0.050

All data are presented as the median (range). The difference between the two groups was compared using the Mann-Whitney U test, with a significance level of *p* < 0.05

### Cells, villous explant culture, and siRNA transfection

The immortalized human trophoblast cell lines, HTR8/SVneo (American Type Culture Collection, Manassas, VA, USA), were cultured in RPMI-1640 (Gibco, Carlsbad, CA, USA) medium supplemented with 10 % fetal bovine serum (FBS; Gemini, California, USA) under standard conditions (37 °C, 5 % CO_2,_ humidified atmosphere). si-ACTN4 (50 nM, 5′-GUUCAUCGUCCAUACCAUC-3′ [22]) and a negative control siRNA were synthesized by GenePharma, Inc. (Shanghai, China). The siRNAs were used to transfect the HTR8/SVneo cells with Lipofectamine™ 3000 (Invitrogen, Carlsbad, CA, USA) according to the manufacturer’s instructions. The collected first trimester villi were sectioned at 2–5 mm, seeded into 24-well plates, precoated with Matrigel (1 µg/µL), and then cultured with DMEM/F12 medium containing 10 % FB before transfection with 500 nM si-ACTN4. The explant outgrowth on the Matrigel was recorded using the EVOS FL Color Imaging System (Thermo Fisher Scientific, Waltham, USA).

### Isolation of primary cytotrophoblast cells

CTBs were isolated from human term placentas as previously described [23]. Briefly, the placental tissues were enzymatically digested through incubation with dispase (Worthington, Lakewood, NJ, USA), 0.25 % trypsin (Gibco), and 0.02 % DNase (Sigma, Missouri, USA) in a 37 °C water bath for 45 min in a way to remove the outer syncytium layer and release the underlying CTBs. Cells were purified via centrifugation at 1400 rpm for 30 min on a 40 % Percoll gradient (GE Healthcare Biosciences, USA) that had been diluted in 10 % 10×HBSS buffer (diluted in ddH_2_O) and pre-centrifuged at 15,000 rpm for 50 min. The cytotrophoblast layer was just above the red blood cell layer. The isolated CTBs were incubated at 37 °C, 5 % CO_2_ for 3 h to allow for cell adhesion or for 48 h to allow for cell syncytialization.

### Immunohistochemistry

The tissue samples were fixed with 4 % paraformaldehyde at room temperature and embedded in paraffin before sectioning at 4 μm. The obtained sections were then deparaffinized in xylene, rehydrated in a serial ethanol gradient, and blocked with 3 % H_2_O_2_ for 10 min. Antigen retrieval was performed through microwaving in 10 mM citric sodium (pH 6.0) for 15 min. Subsequently, the sections were incubated with a rabbit polyclonal primary antibody against ACTN4 (1:200, Proteintech, Wuhan, China), a mouse monoclonal antibody against CK7 (1:100, Abcam, Cambridge, UK), or a mouse monoclonal antibody against HLA-G (1:100, Proteintech) overnight at 4 °C. The secondary antibodies conjugated to horseradish peroxidase were then added for 30 min at room temperature, followed by additional incubation with DAB solution for visualization. The sections were subsequently counterstained with haematoxylin (Nanjing JianCheng Bioengineering Institute, Nanjing, China).

### Immunofluorescence staining

Cells were fixed with 4 % paraformaldehyde, permeabilized in 0.2 % Triton X-100, and then incubated with the indicated antibodies overnight at 4 °C prior to further incubation with FITC-conjugated or Cy3-conjugated goat anti-rabbit or mouse fluorescent antibodies (Proteintech, 1:100) at room temperature for 1 h. For actin filament staining, permeabilized cells were incubated with 100 nM TRITC-phalloidin (Solarbio, Beijing, China) solutions directly for 30 min at room temperature. The nuclei were stained with DAPI, and images were visualized using the EVOS FL Color Imaging System (Thermo Fisher Scientific).

### Western blotting

Protein extracts were prepared from the tissues and cells using a RIPA lysis buffer supplemented with PMSF (Beyotime Institute of Biotechnology, Jiangsu, China), protein concentrations were measured using a bicinchoninic acid (BCA) Protein Assay kit (Beyotime). Equal amounts of protein were separated by SDS-polyacrylamide gel electrophoresis (Bio-Rad Laboratories, Hercules, California, USA), and then transferred onto PVDF membranes (Millipore, Darmstadt, Germany). Following blocking with 5 % nonfat dry milk (Bio-Rad) at room temperature for 1 h, the membranes were immunoblotted overnight at 4 °C with primary antibodies against ACTN4 (1:3000, Proteintech), GCM-1(1:500, Proteintech), AKT (1:1000, Abcam), AKT phosphorylated at serine 473 (1:1000, Cell Signaling Technology, Danvers, MA, USA), p-GSK3β (1:1000, Cell Signaling Technology), Snail (1:1000, Cell Signaling Technology), N-cadherin (1:1000, Cell Signaling Technology), Vimentin (1:1000, Abcam), or β-actin (1:1000, Proteintech). After the membranes were rinsed with TBST, another incubation with the respective HRP-conjugated secondary antibodies (1:5000, Zhong San Golden Bridge Crop, Beijing, China) was carried out at room temperature for 1 h. Immunoreactive bands were detected using an enhanced chemiluminescent substrate (Millipore), and the images were captured and analyzed using the ChemiDoc XRS + system (Bio-Rad).

### Matrigel invasion and transwell migration assay

The invasion ability was measured using a transwell chamber consisting of a 24-well plate with membrane inserts (Corning, New York, USA) containing polycarbonate filters (pore size: 8 μm) that were precoated with 60 µL of 1 mg/mL Matrigel matrix solution (BD Biosciences, California, USA). A suspension of 8 × 10^4^ cells in 200 µL of serum-free culture medium was added to the inserts, which were then placed in the lower chamber containing 600 µL of 10 % FBS culture medium. The cells were left to migrate through the membranes for 24 h prior to fixing with 4 % paraformaldehyde and staining with crystal violet. Images were captured using the EVOS FL Color Imaging System (Thermo Fisher Scientific). The cell migration assay was performed in the same manner, except that the inserts were not coated with Matrigel.

### EdU proliferation assay

A total of 100 µL of EdU culture medium (1:1000, 50 µM) was added to each well of the villous explants for 12 h, followed by fixing with 4 % formaldehyde for 30 min, paraffin embedding, and sectioning at 4 μm. After intensive washing, the tissue sections were subjected to a Click-iTR EdU Kit (RiboBio, Guangzhou, China) for 30 min and stained with 100 µL of DAPI (RiboBio) at room temperature for 30 min. Image analysis was performed on a fluorescence microscope (EVOS FL Color Imaging System). The relative percentage of EdU-positive cells was determined by examining three to five samples in three wells.

## Wound‐healing assay

Cells were grown to confluence on a 6-well plate and then mechanically scratched with a sterile pipette tip. After removal of the non-adherent cells via rinsing with PBS, the remaining cells were grown in serum-free 1640 medium with ACTN4 siRNA for an additional 24 h. The cell motility, i.e., wound closure, was measured by photographing three random fields at the 0 and 24 h time points.

### Statistical analysis

Data are presented as the means ± SEM. Statistical data were analyzed using Student’s *t*-tests (a significance level of *p* < 0.05). All statistical analyses were performed using GraphPad Prism 7 software (La Jolla, California, USA).

## Results

### ACTN4 was highly expressed in the CTBs of placental villi

**Fig. 1:**
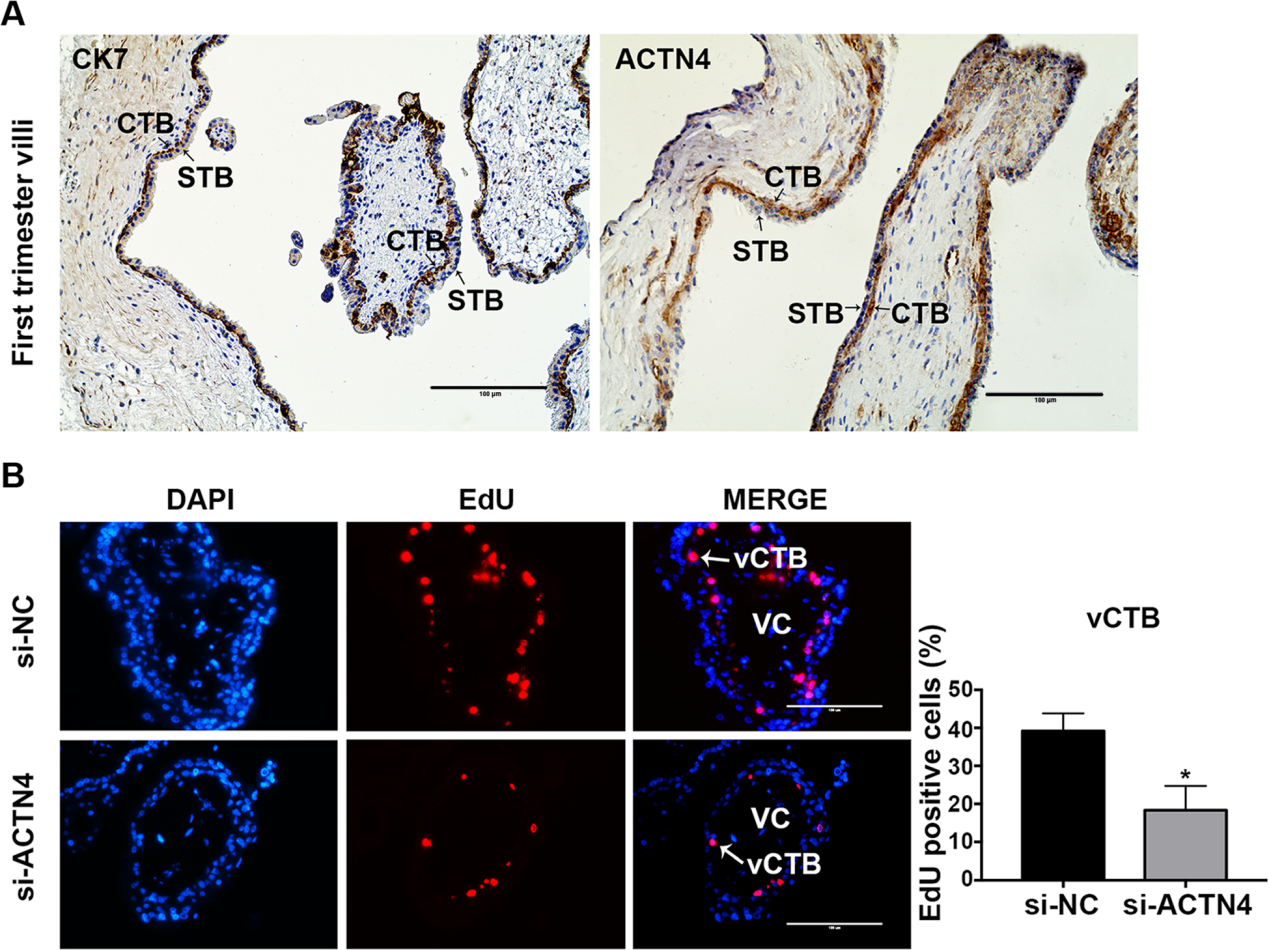
ACTN4 expression in placental CTBs. **a** IHC staining of ACTN4 in the human first trimester placenta villi. CTBs were stained for CK7; Scale bars: 100 μm. **b** EdU labeling in explant cultures. Villous explants isolated from the first trimester placentas were treated with the control siRNA (si-NC) or ACTN4 siRNA (si-ACTN4). Representative pictures showed EdU (pink) labeling in the villous cytotrophoblasts of floating villi following 72-h treatment. Nuclei were counterstained with DAPI (blue). Scale bars: 100 μm. All data are presented as the means ± SEM of three independent experiments. ^*^p < 0.05

Following the idea that ACTN4 may have an ever-emerging role in human placentation, we first detected the expression pattern of ACTN4 in the first trimester villi by immunohistochemistry and identified CTBs with the use of an anti-CK7 antibody. The results showed that ACTN4 was strongly expressed in the CTBs of the first trimester villi, while no obvious staining was detected in the STBs (Fig. 1 A). Owing to the recent finding that CTB proliferation and trophoblast stemness are responsible for early placental development [24, 25], we next explored the role of ACTN4 in trophoblast proliferation. This was accomplished by treatment of the first trimester villous explants with a siRNA that specifically targeted the mRNA of ACTN4. Immunofluorescence analyses revealed that ACTN4 downregulation reduced EdU labeling of the CTBs in floating villous explants (Fig. 1B). These data suggested that ACTN4 may be involved in trophoblast proliferation.

### ACTN4 was downregulated during the differentiation of CTBs into STBs

**Fig. 2:**
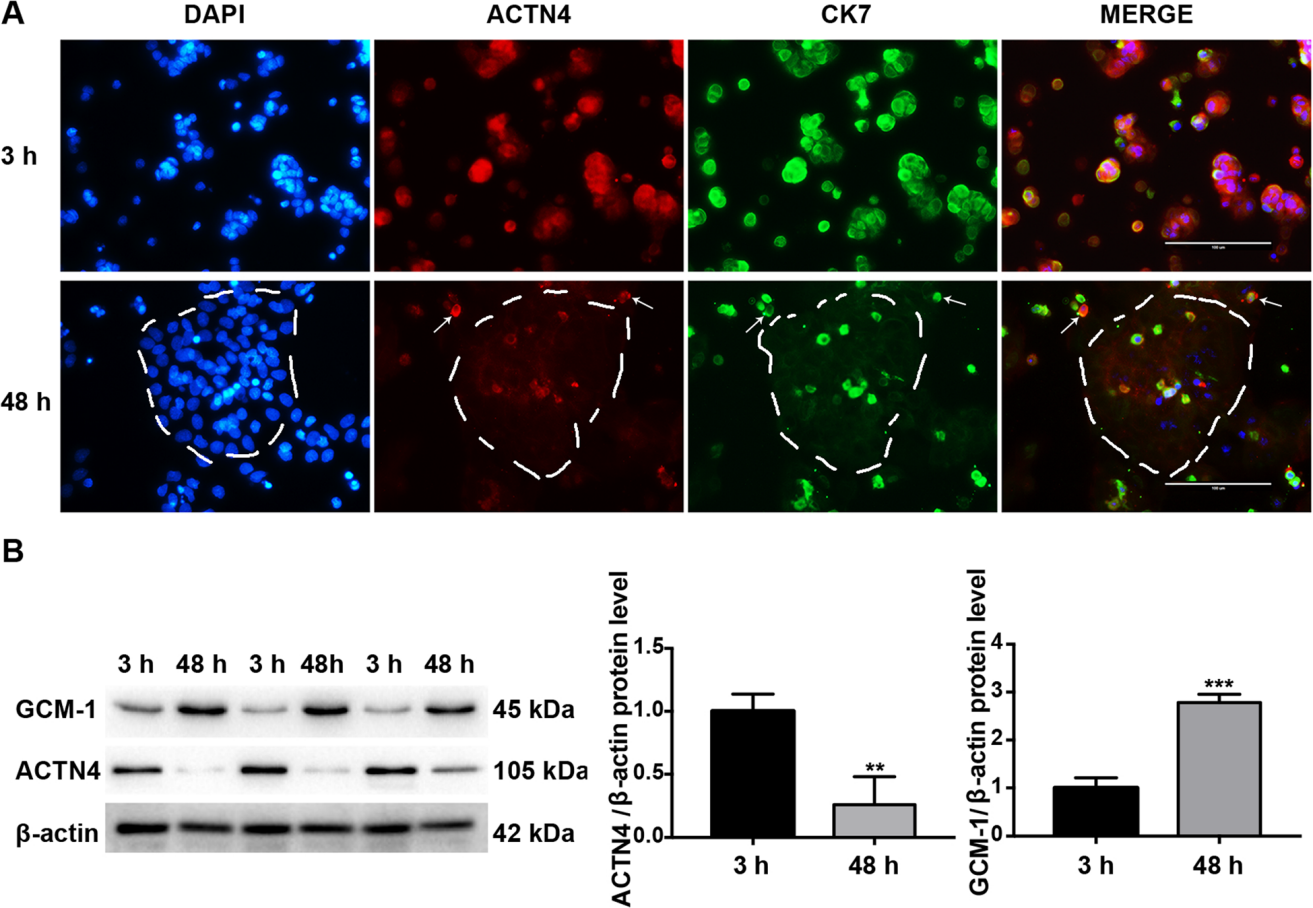
ACTN4 expression in the process of CTB differentiation into STBs. **a** Immunofluorescence staining for ACTN4 (red) and CK7 (green) in the freshly isolated primary human CTB cells. Staining was performed after 3 or 48 h of culture. Nuclei were counterstained with DAPI (blue). White dashed circles and white arrows indicate syncytialized cells and unsyncytialized cells, respectively. Scale bars: 100 μm. **b** Western blotting for ACTN4 and GCM-1 (a syncytiotrophoblast marker) in the primary human CTB cells after 3 or 48 h of culture. All data are presented as the means ± SEM of three independent experiments. ^**^P < 0.01; ^***^P < 0.001

STBs arise by fusion of the CTBs with proliferative properties, and DNA synthesis is found absent in the human STB cells [26], demonstrating that the differentiation from CTBs into STBs occurs simultaneously with the loss of cell proliferation abilities. Our above-mentioned finding that downregulation of ACTN4 decreased trophoblast proliferation revealed that CTB differentiation into STBs may be accompanied by a reduced ACTN4 expression. To test this hypothesis, we isolated the primary CTBs from term placentas and allowed them to differentiate into STBs in a gradual, spontaneous manner during 48 h of *in vitro* culture. The results showed that ACTN4 was strongly stained and expressed in the primary CTBs but barely detected during the course of differentiation (Fig. 2), suggesting that ACTN4 was downregulated in the CTB loss of proliferative abilities. Taken together, these data further confirmed that ACTN4 may play a pivotal role in CTB proliferation.

### ACTN4 deficiency reduced proliferation of cell column trophoblasts and outgrowth of extravillous explants

**Fig. 3:**
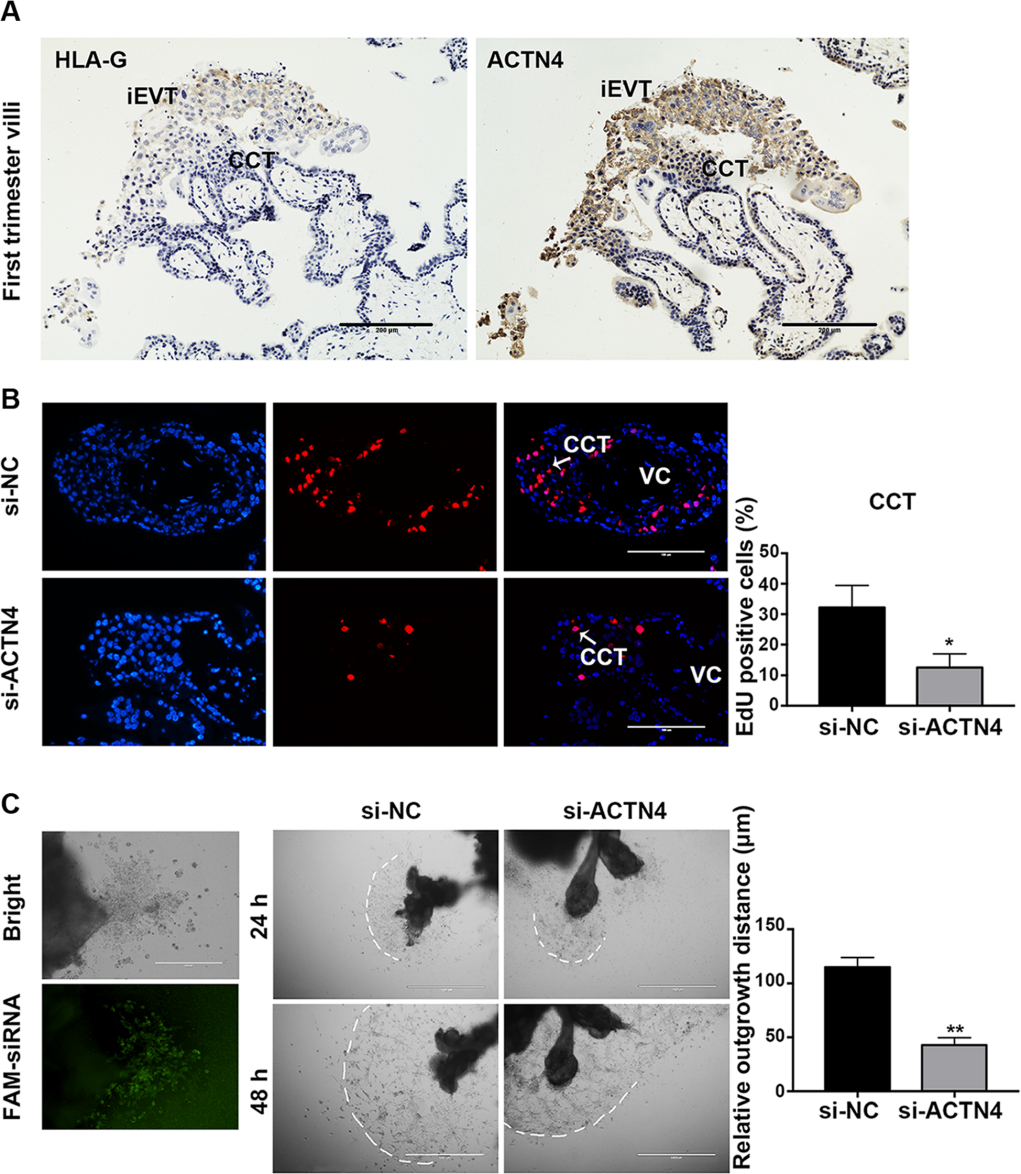
Role of ACNT4 in villous explants outgrowth. **a** IHC staining of ACTN4 in the human first trimester placenta villi. iEVTs were stained for HLA-G. Scale bars: 200 μm. **b** EdU labeling in explant cultures. Representative pictures showed EdU (pink) labeling in the CCTs of the anchoring villi after 72-h treatment of ACNT4 with siRNA. Nuclei were counterstained with DAPI (blue). Scale bars: 100 μm. **c** Outgrowth of induced EVTs in a villous explant culture model. Left panel, bright and fluorescent field views showing that small interfering RNA labeled with FAM successfully penetrated the villous explants. Right panel, the outgrowth of induced EVTs from villous explants treated with control siRNA (si-NC) or ACTN4 siRNA (si-ACTN4) for 24 and 48 h. Scale bar: 1000 μm. All data are presented as the means ± SEM of three independent experiments, ^*^P < 0.05; ^**^P < 0.01

In addition to the high expression in CTBs, ACTN4 was also strongly stained in the HLA-G-labeled CCTs in the first trimester villi, with an increased expression level spotted at the distal region where the cells transformed into iEVTs after undergoing an EMT process (Fig. 3 a). Immunofluorescence analyses revealed that the knockdown of ACTN4 led to a declined EdU labeling of CCTs in the anchoring villous explants (Fig. 3b). Moreover, inhibited ACTN4 was found to reduce the outgrowth distance of Matrigel-seeded villous explants (Fig. 3 c). These results demonstrated that loss of ACTN4 impedes the outgrowth of villous explants by reducing CCT proliferation.

### Inhibition of ACTN4 decreased HTR8/SVneo cell invasion and migration

**Fig. 4:**
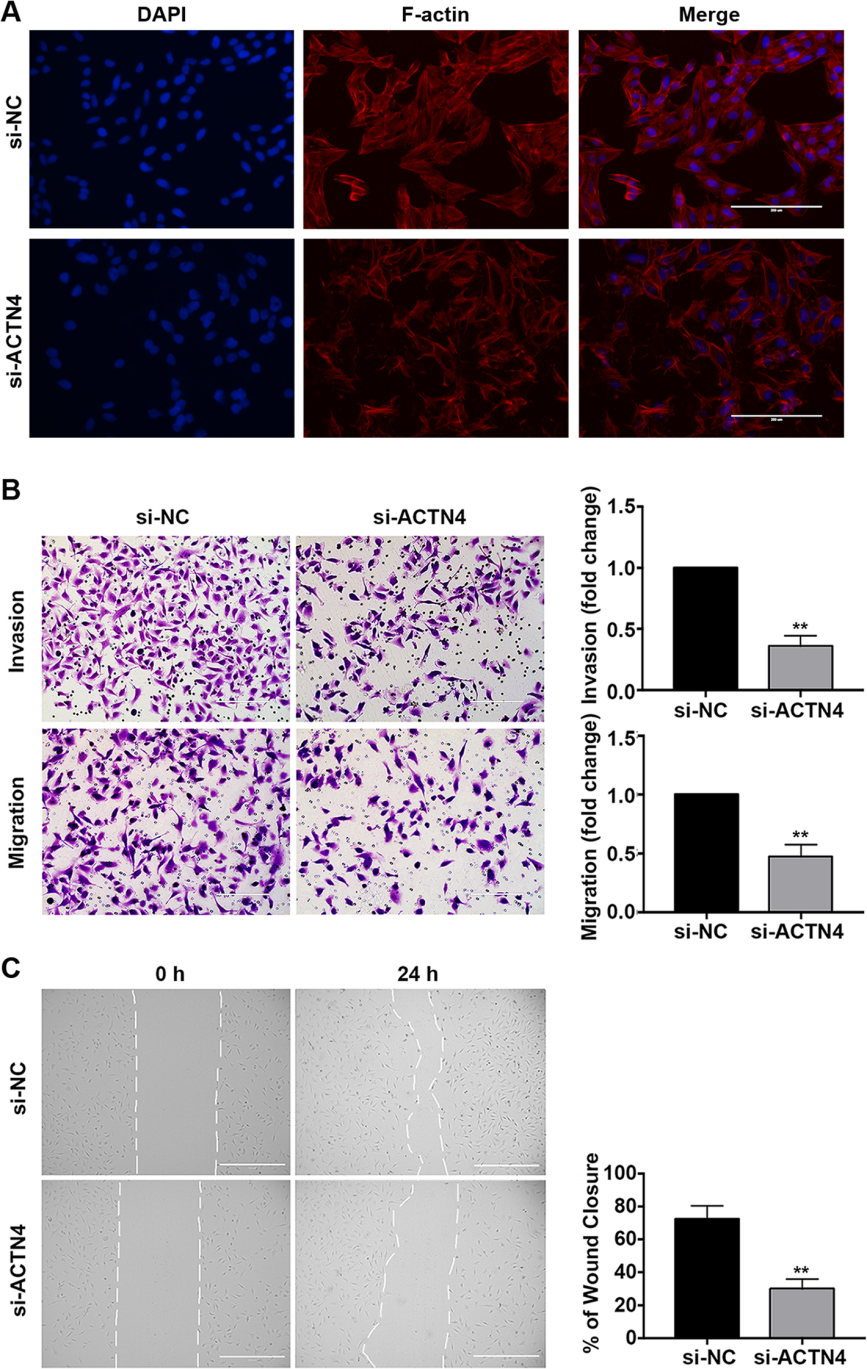
Effects of ACTN4 on HTR8/SVneo cell invasion and migration. **a** TRITC-Phalloidin staining on si-NC and si-ACTN4 of HTR8/SVneo cells. Scale bar, 200 μm. **b** Representative images and quantification of cells from the HTR8/SVneo cells with invasion in the presence or absence of a Matrigel-coated membrane for 24 h. Scale bar: 200 μm. **c** HTR8/SVneo cells attached to the culture plates after wounding and treatment with ACTN4 siRNA. The percentage of wound closure was calculated at 24 h. Scale bar: 400 μm. All data are presented as the means ± SEM of three independent experiments. ^**^P < 0.01

Since ACTN4 is highly abundant in iEVTs and the major function of the latter is to migrate and invade into the maternal decidua [27], we proceeded to verify whether ACTN4 has a significant role in cell invasion and migration. First, TRITC-phalloidin staining showed that the organization of actin filaments (F-actin) was disrupted in ACTN4 silenced of HTR8/SVneo cell (Fig. 4 a). Then, the Matrigel cell invasion and transwell cell migration assay results showed that ACTN4-deficient cells displayed lower invasion and migration abilities than the control cells (Fig. 4b). Furthermore, wound-healing assays confirmed that the knockdown of ACTN4 markedly inhibited cell migration (Fig. 4 c). These findings altogether indicated that ACTN4 deficiency attenuates iEVT invasion and migration during placentation.

### AKT activation and Snail expression were involved in the ACTN4-induced proliferation, invasion, and migration of trophoblasts

**Fig. 5:**
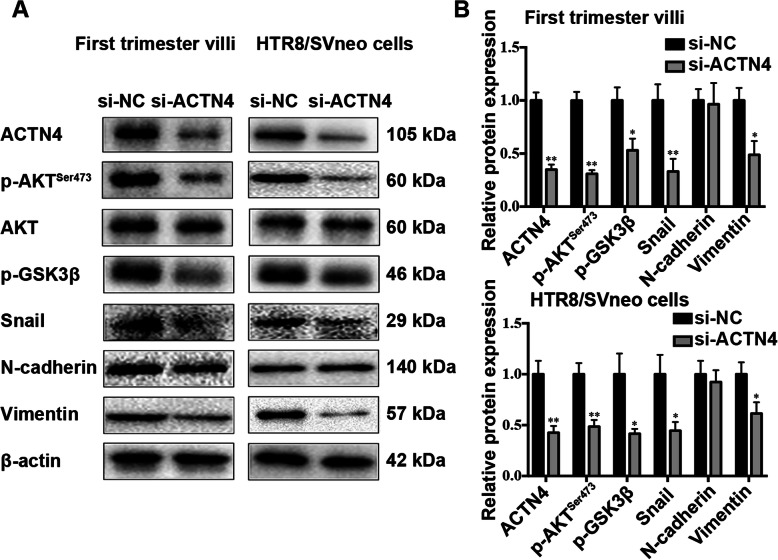
ACTN4 regulation of the AKT/GSK3β/Snail pathway in trophoblasts. Western blotting for ACTN4, p-AKT^Ser473^, AKT, p-GSK3β, Snail, N-cadherin, and Vimentin in the first trimester villi (left panel) and HTR-8/SVneo cells (right panel) transfected with si-NC or si-ACTN4 for 72 h. All data are presented as the means ± SEM of three independent experiments. ^*^P < 0.05; ^**^P < 0.01

To investigate the regulatory mechanism of ACTN4 in trophoblast function, we first examined the role of ACTN4 in regulating the AKT pathway in the first trimester villi. Western blot analyses suggested that ACTN4 knockdown reduced AKT phosphorylation at Ser473; accordingly, the phosphorylation level of its downstream effector GSK3β was also remarkably declined (Fig. 5). Moreover, ACTN4 silencing inhibited the expression level of Snail, a transcription factor that potentiates the EMT process, and an evident reduction was also noted in the Snail-modulated mesenchymal marker vimentin (Fig. 5). Whereas, another mesenchymal marker, N-cadherin, had no significant expression alterations in the ACTN4 knockdown group. The same results were also observed for the HTR8/SVneo cells. From these data, we concluded that ACTN4 might regulate trophoblast proliferation, invasion, and migration through the AKT/GSK3β/Snail pathway.

### ACTN4 was hardly detectable in the iEVTs of sPE placentas

**Fig. 6:**
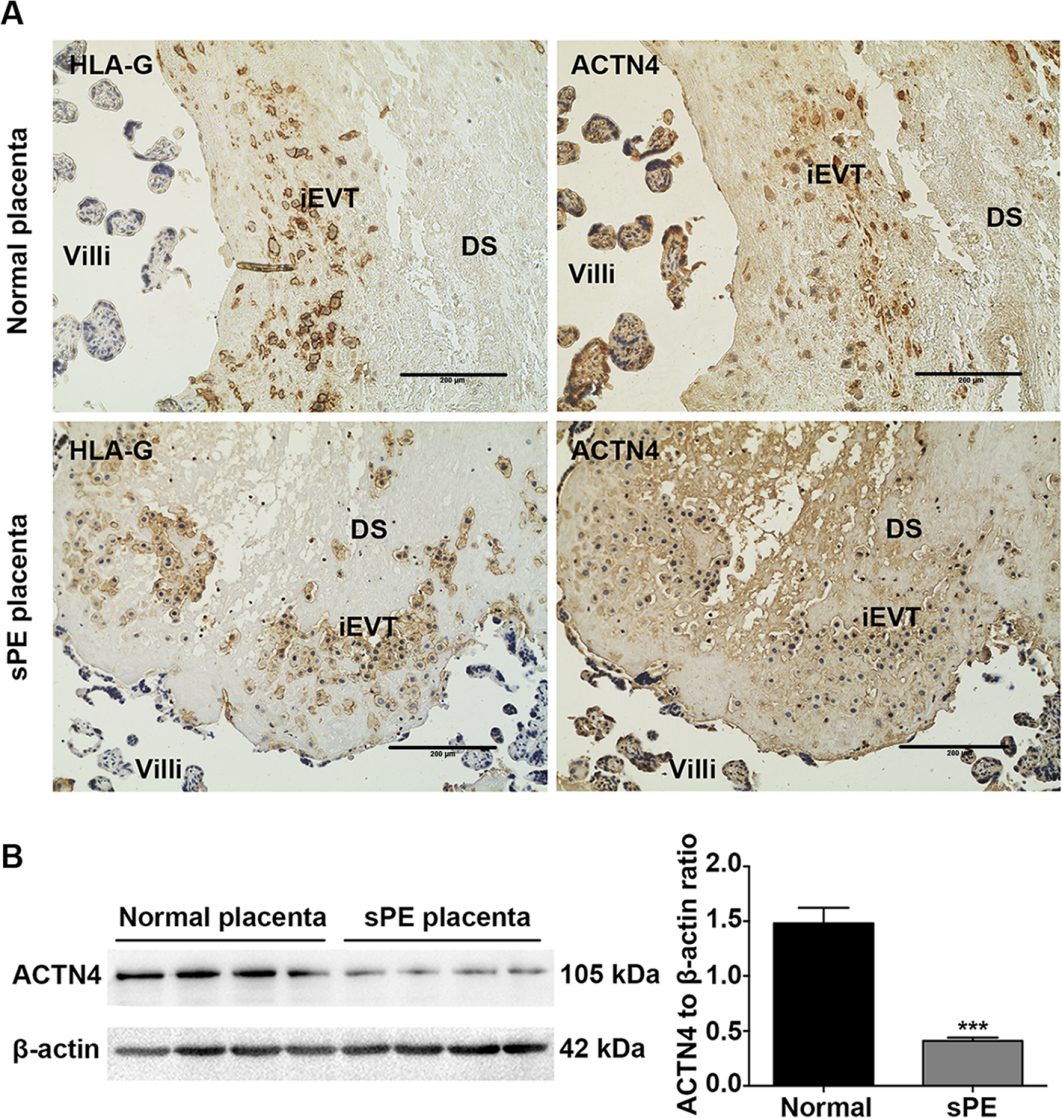
ACTN4 expression in the iEVTs from sPE placentas. **a** IHC staining of ACTN4 on the maternal side of normal and sPE placentas. Staining was performed on serial sections; iEVTs were stained for HLA-G. DS: decidual side. Scale bars: 200 μm. **b** Western blotting for ACTN4 on the maternal side of normal and sPE placentas. All data are presented as the means ± SEM of three independent experiments. ^***^P < 0.001

Shallow invasion of iEVTs into the maternal decidua has been widely recognized as the primary cause of PE development [6]. In this context, we proceeded to examine the expression pattern of ACNT4 in the iEVTs of normal and sPE placentas. IHC assays showed that ACTN4-positive staining was observed in the HLA-G-labeled iEVTs on the maternal side of full-term normal placentas but barely detected in the sPE placentas (Fig. 6 a). Subsequently, we compared the expression level of ACTN4 in those placentas by Western blotting. Interestingly, the ACTN4 level was found significantly lower in the sPE placentas than in the normal controls (Fig. 6b).

## Discussion

Proper development of the placenta and its component lineages at early stages is critical for successful pregnancy [28]. Dysregulation of EVTs disrupts the normal invasion of trophoblasts into the uterus, which consequently leads to incomplete spiral artery remodeling and placental hypoperfusion [29]. In the current study, ACTN4 was predominantly expressed in the CTBs and iEVTs from normal placentas but barely detected in those cells from sPE placentas. Downregulation of ACTN4 reduced villous trophoblast proliferation and explant outgrowth *ex vivo*. In addition, ACTN4 deficiency resulted in significant inhibition of cell invasion and motility. Such attenuated proliferation, invasion, and migration was a result of ACTN4-mediated inactivation of the AKT/GSK3β/Snail pathway. CTBs are the so-called epithelial stem cells of the placenta, which, depending on the signals they receive, can maintain the balance between their differentiation into either STBs or EVTs [30]. Additionally, isolated CTBs without proliferative capabilities can spontaneously differentiate into STBs following 24-h culture [31], suggesting that the self-renewal potential of CTBs are essential for maintaining the proliferation and differentiation abilities. Our IHC data and primary CTB culture studies revealed that ACTN4 was highly expressed in CTBs but not in STBs, and a downregulated ACTN4 expression level reduced CTB proliferation and explant outgrowth, indicating that ACTN4 deficiency may facilitate STB fusion but is detrimental to EVT development. As previously reported [32], ACTN4 is a functional partner of AKT, and thus ACTN4 silencing inhibits AKT membrane translocation and phosphorylation, which results in a significant reduction in cell proliferation. Moreover, ACTN4 downregulation contributed to diminished AKT phosphorylation in both villous explants and HTR8/SVneo cells. Based on these observations, we believe that ACTN4 exerts an impact on CTB proliferation by modulating AKT activation.

Although ACTN4 is frequently expressed in the distal region of CCTs and iEVTs, the exact role of ACTN4 in CCTs undergoing EMT remains unknown. In the present study, we found that ACTN4 knockdown decreased AKT and GSK3β phosphorylation and the expression of Snail. Snail has a pivotal part in the modulation of EMT and is involved in tumorigenesis via repression of epithelial marker proteins and induction of mesenchymal marker proteins [33, 34]. Our results revealed that ACTN4 downregulation impeded EMT progression by suppressing the expression of EMT-associated proteins, such as Snail and Vimentin, resulting in abnormal trophoblast invasion and migration. Additionally, the EMT process includes cytoskeleton remodeling [35], and ACTN4 is a cytoskeletal protein that is primarily localized in the filopodia extension and involved in the extension of podosomes during cell invasion and migration [36, 37]. Moreover, ACTN4 dysregulation leads to the disassembly and disruption of actin filament-based structures, such as filopodia and podosomes [38–40]. This evidence indicated that ACTN4 silencing may lead to the disorganization of actin filaments via the EMT approach, and, ultimately, impaired filopodia and podosome formation result in inadequate EVT invasion and migration.

## Conclusions

Our findings suggest that the appropriate expression of ACTN4 is essential for normal proliferation and differentiation of CTBs during early pregnancy. ACTN4 downregulation may result in inadequate trophoblast proliferation, invasion, and migration through the mediation of the AKT/GSK3β/Snail pathway. Besides, dysregulation of this protein may contribute to preeclampsia.

## Data Availability

All data generated through this study are included in this article.

## Abbreviations

CTB: cytotrophoblasts
STB: syncytiotrophoblast
EMT: epithelial-to-mesenchymal transition
EVT: extravillous trophoblasts
ACTNs: Alpha-actinins
IHC: Immunohistochemistry
